# Sleep Apnea Detection Using Multi-Error-Reduction Classification System with Multiple Bio-Signals

**DOI:** 10.3390/s22155560

**Published:** 2022-07-25

**Authors:** Xilin Li, Frank H. F. Leung, Steven Su, Sai Ho Ling

**Affiliations:** 1 School of Biomedical Engineering, Faculty of Engineering and Information Technology (FEIT), University of Technology Sydney (UTS), Ultimo, NSW 2007, Australia; xilin.li@alumni.uts.edu.au (X.L.); steven.su@uts.edu.au (S.S.); 2 Department of Electronic and Information Engineering, The Hong Kong Polytechnic University, Hung Hum, Hong Kong, China; frank-h-f.leung@polyu.edu.hk; 3 School of Electrical and Data Engineering, Faculty of Engineering and Information Technology (FEIT), University of Technology Sydney (UTS), Ultimo, NSW 2007, Australia

**Keywords:** feature extraction, feature selection, polysomnography, sleep apnea detection

## Abstract

Introduction: Obstructive sleep apnea (OSA) can cause serious health problems such as hypertension or cardiovascular disease. The manual detection of apnea is a time-consuming task, and automatic diagnosis is much more desirable. The contribution of this work is to detect OSA using a multi-error-reduction (MER) classification system with multi-domain features from bio-signals. Methods: Time-domain, frequency-domain, and non-linear analysis features are extracted from oxygen saturation (SaO${}_{2}$), ECG, airflow, thoracic, and abdominal signals. To analyse the significance of each feature, we design a two-stage feature selection. Stage 1 is the statistical analysis stage, and Stage 2 is the final feature subset selection stage using machine learning methods. In Stage 1, two statistical analyses (the one-way analysis of variance (ANOVA) and the rank-sum test) provide a list of the significance level of each kind of feature. Then, in Stage 2, the support vector machine (SVM) algorithm is used to select a final feature subset based on the significance list. Next, an MER classification system is constructed, which applies a stacking with a structure that consists of base learners and an artificial neural network (ANN) meta-learner. Results: The Sleep Heart Health Study (SHHS) database is used to provide bio-signals. A total of 66 features are extracted. In the experiment that involves a duration parameter, 19 features are selected as the final feature subset because they provide a better and more stable performance. The SVM model shows good performance (accuracy = 81.68%, sensitivity = 97.05%, and specificity = 66.54%). It is also found that classifiers have poor performance when they predict normal events in less than 60 s. In the next experiment stage, the time-window segmentation method with a length of 60 s is used. After the above two-stage feature selection procedure, 48 features are selected as the final feature subset that give good performance (accuracy = 90.80%, sensitivity = 93.95%, and specificity = 83.82%). To conduct the classification, Gradient Boosting, CatBoost, Light GBM, and XGBoost are used as base learners, and the ANN is used as the meta-learner. The performance of this MER classification system has the accuracy of 94.66%, the sensitivity of 96.37%, and the specificity of 90.83%.

## 1. Introduction

Obstructive sleep apnea (OSA) is the most common breathing disorder during sleeping, which is caused by repeated partial or total obstruction of the upper airway [1]. The obstruction of the upper airway may last for 10 s or more. OSA has a risk for several complications, such as hypertension and cardiac diseases. The number of apneas and hypopneas in one hour during sleep (apnea–hypopnea index, AHI) diagnosis the severity of OSA. Clinically, for diagnosing OSA, polysomnography (PSG) is considered the gold standard. PSG records overnight electrocardiogram (ECG), electromyogram (EMG), electrooculogram (EOG), electroencephalogram (EEG), airflow, respiratory effort, oxygen saturation (SaO${}_{2}$), body position, and snore signals. The diagnosis of OSA by trained specialists requires expensive human resources and relies on the experience level of a specialist. It is a time-consuming and difficult task for expert physicians to analyse a large volume of data that is collected overnight. Thus, an automatic OSA detection system is desirable to help doctors during a diagnostic process.

Recently, studies on automatic detection methods using PSG signals were reported. The recent work had shown that SaO${}_{2}$ signals provided discriminative information in sparse representation to detect apnea–hypopnea events [2]. Systematic and sporadic noise were removed from airflow signals using the sliding window and short-time slice methods. Then, a Bayesian criterion is used to classify apnea or hypopnea events [3]. Vaquerizo-Villar et al. [4] used anthropometric variables, time-domain features, and spectral features from SaO${}_{2}$ recordings to classify apnea from the normal events using the multi-layer perceptron. In [5], unlabelled ECG signals provided features obtained by the sparse auto-encoder method, and an artificial neural network (ANN) was employed to detect OSA. Then, the hidden Markov model was used to improve the performance. An optimised convolution neural network structure was used to develop the apnea event, and SaO${}_{2}$ signals were used [6]. In Study [7], the authors proposed a new probabilistic algorithm. A Gaussian mixture probability model was used to detect apnea events based on the posterior probabilities of the respective events. In Study [8], the authors used a hybrid feature selection method to obtain multi-domain significant features from ECG, SaO${}_{2}$, and abdominal signals. Mahsa Bahrami [9] used ECG recordings and hybrid deep models to achieve high performance. Yao Wang [10] designed a single-channel EEG sleep monitoring model based on the bidirectional long and short-term memory network (the accuracy of 92.73%).

The four contributions of this paper are as follows. First, we extracted new time-domain, frequency-domain, and non-linear features from multiple bio-signals. Second, we designed a two-stage feature selection to select a feature subset. Third, for practical reasons, we proposed a time-window method and combined it with classifiers to achieve good results. Fourth, we designed a multi-error-reduction (MER) classification system that utilises the proposed feature extraction and selection to improve classification performance.

This paper is organised as follows. Section 2 discusses the general structure, dataset, feature extraction methods, two-stage feature selection, and some classification algorithms. Section 3 reports results with discussions. A conclusion will be drawn in Section 4.

## 2. Methodology

### 2.1. General Structure

**Figure 1:**
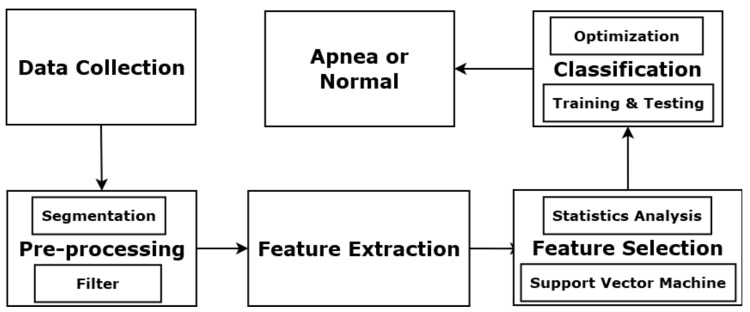
Components of OSA detection system.

Figure 1 illustrates the basic components of the proposed OSA detection system. The process starts with PSG signal collection, followed by a signal pre-processing module with segmentation and filtering. Then, a feature extraction module obtains features from the PSG signals. Next, a feature selection module determines a useful feature subset. Finally, the selected features are added to the MER classification system. Each input event can be labelled as normal or apnea.

### 2.2. Collection of Sleep Studies from a Database

**Figure 2:**
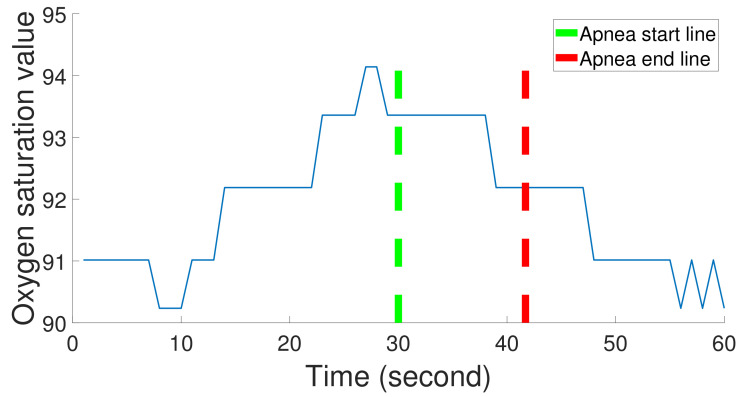
One apnea event duration in SaO${}_{2}$ signals; the duration is between the apnea start line and the apnea end line.

To construct our apnea classification system, we use the Sleep Heart Health Study (SHHS) database provided by the National Heart Lung and Blood Institute. The dataset covers people over 40 years old and consists of thoracic, abdominal, airflow, SaO${}_{2}$, and ECG signals. Thoracic and abdominal excursions are recorded by inductive plethysmography bands and sampled at 10 Hz. The bands are placed on a subject’s thorax and abdomen. SaO${}_{2}$ signals are collected by a finger-tip pulse oximetry and sampled at 1 Hz. Airflow signals are collected by a nasal-oral thermocouple and sampled at 10 Hz. The airflow sensors are placed under a subject’s nose. ECG is collected by a bipolar lead, sampled at 125 Hz, with two leads placed R subclavicle-L lower rib. Heart rate (RR intervals) signals are derived from ECG signals sampled at 1 Hz. A sleep physiologist labels each apnea event, and its start and end points. Figure 2 shows SaO${}_{2}$ signals, an apnea event, and its start and end points. In this study, ECG, abdominal, thoracic, airflow, and SaO${}_{2}$ signals are used.

#### 2.2.1. Feature Extraction Using SaO${}_{2}$ Signals

We use 12 multi-domain extraction methods to obtain features from the SaO${}_{2}$ signals. They are features 1–12 in Table 1. The mostly used index for diagnosing OSA is the cumulative time that lasts below a threshold. As a preprocessing of the data, non-physiological artifacts are considered as artifacts when the point-to-point difference is more than 8%, and the median value is calculated for the beginning 10 s to replace the artifacts.

In this study, the feature set covers multi-domain features that include the median of each window (med), the maximum-to-minimum changes which were more than 2% of each window (MM2), the kurtosis of each window (kur), the variance of each window (var), the minimum of each window (min), the mean of each window (mean), the number of zero-cross in the window (NumZC), and the complexity of each window (comp). Complexity represents the length of the shortest description in an SaO${}_{2}$ window. The Poincare SD${}_{1}$ is computed, which shows the short-term variability of each SaO${}_{2}$ window [11]. Two features are the time spent below and above the 98% maximum in each window (Bel98 and Abo98). Finally, the power spectral density (PSD) method is used to show the intensity of desaturation events. A 5th-order Yule–Walker autoregressive estimate is used to obtain the mean of PSD in the 0.016–0.05 Hz frequency band in each window (mean_PSD${}_{0.016/0.05}$).

#### 2.2.2. Feature Extraction Using Airflow Signals

Airflow recordings provide features 13 to 22 shown in Table 1. The apnea index from airflow signals is related to a decrease to at least 10% of its basal value that sustains for at least 10 s [12]. The apnea events are scored for at least two missed breaths [13]. To preprocess that data, we obtain the median of a 10 s window, which is used to correct the baseline. For noise removal, a 3rd-order Butterworth low-pass filter with a cut-off frequency of 3 Hz is used. Airflow recordings are re-sampled at 1 Hz.

In each window, there are statistical features, including the mean (mean), median (med), and standard deviation (std). On the other hand, the decreases and increases of airflow signals throughout the night are related to the frequency domain. PSD and the wavelet algorithm are used to explore the differences in the spectral information between the sleep apnea positive and negative groups. The Welch method uses a segment length of five samples with 2.5 overlapped samples. The mean within the frequency ranges of 0–0.1 Hz and 0.4–0.5 Hz in each window is calculated (mean_PSD${}_{0/0.1}$ and mean_PSD${}_{0.4/0.5}$). The depth of wavelet transformation is three, and Daubechies wavelets three is used. The wavelet can decompose a given window signal to obtain means of one approximation level and three detail coefficient levels (mean_D1 to mean_A3). Finally, there is a variable called complexity in each window (comp).

#### 2.2.3. Feature Extraction Using Abdominal and Thoracic Signals

We use time-domain and frequency-domain methods to process abdominal and thoracic recordings. Features 23–28 are extracted from the abdominal recordings, and Features 29–34 are extracted from the thoracic recordings, as shown in Table 1. The collapse of the upper airway leads to OSA, which causes activities of the respiratory muscles. For abdominal and thoracic recordings, we use the median of a 60 s window to start the baseline correction. A Butterworth filter with a pass-band of 0.05–4 Hz is then used to remove noise.

The feature set includes the summation and the standard deviation of each absolute window (sum_abs and std_abs) and the mean of each window (mean). The Yule–Walker method and wavelet transformation are used to extract frequency-domain features. The segmentation length of the Yule–Walker method is 40 samples, and the mean of the 80–100 Hz frequency range in each window (mean_PSD${}_{80/100}$) is obtained. The wavelet is Daubechies 2 with a depth of two, and the mean of the first and second detail levels are computed (mean_D1 and mean_D2) in each window. Next, features in the frequency domain and time domain are extracted from thoracic recordings. This feature set consists of the median, summation, mean, variance, and standard deviation of each window (sum, med, std, mean, var). The summation of the 80–100 Hz frequency band (sum_PSD${}_{80/100}$) in each window is computed by the Yule–Walker method.

#### 2.2.4. Feature Extraction Using ECG Signals

In this study, three kinds of feature extraction methods are used to obtain features from ECG signals. Table 1 shows them as features 35–66. To process the data, first, the 0.05–40 Hz band-pass thirrd Butterworth filter is applied to remove noise and takes baseline correction. Then, the R-peaks are found by the modified Pan–Tompkin algorithm. QRS series are extracted by a symmetric window of 120 ms around the R-peaks. Because of the low ECG quality, there is a processing step to calculate a corrected RR interval sequence. In this work, we use the heart rate correction method from [14]. Finally, ECG-derived respiratory (EDR) signals are obtained from the ECG recordings by the Physionet EDR method since EDR signals can reflect the motion of the thoracic cavity during sleep.

We first obtain features using time-domain methods. These features include the number of pairs of adjacent RR intervals that the later RR interval is more than the previous one by more than 50 ms (NN50_RR), the standard deviation of the RR interval (SDSD_RR), the standard deviation of the RR interval between the standard deviation at the first 30 s and the one at the second 30 second (tSD_RR), the standard deviation of RR interval (std_RR), the variance, the kurtosis of each ECG window (var and kur), the mean of each RR interval window (mean_RR), and a ratio of the standard deviation to the mean of each EDR window (CV_EDR).

Second, we use PSD and wavelet transformation to extract frequency-domain features. In each window, we extract spectral features, such as spectral spread (SS) and spectral decrease (SD). The wavelet transformation with a Symlet wavelet of order three and a level number of seven is used. Shannon’s entropy (entropy_D1 to entropy_D7) and variance (var_D1 to var_D7) are computed using seven detailed coefficient levels. Wavelet spectral density (WSD) is used to analyse the RR series (WSD_RR). PSD is used to process the RR series and ECG signals. In each RR interval window, the dominant frequency is found in the 0.03–0.5 Hz frequency range (max_PSD${}_{0.03/0.5}$). In each ECG window, we extract the mean of PSD in 10–20 Hz and 80–100 Hz (mean_PSD${}_{10/20}$ and mean_PSD${}_{80/100}$).

Finally, two serial correlation coefficients are extracted from each RR interval window (SCrC_3_RR to SCrC_4_RR). Here, the kernel principal component analysis (kPCA) is done on the QRS recordings, and the maximum of the diagonal matrix of kPCA (max_dia_kPCA) and the relative power of the second principal component (RP_2_PC) are calculated in each QRS window.

In summary, we obtain 66 features. Features 1–12 are from SaO${}_{2}$ signals, Features 13–22 are from airflow signals, Features 23–28 are from abdominal recordings, Features 29–34 are from thoracic recordings, and Features 35–66 are from ECG signals, which are shown in Table 1.

### 2.3. Feature Selection

A large number of features increase the processing time, but it might not be needed, as some features could be redundant. Hence, a feature selection is used to remove redundant features before the classification stage, which helps to prevent over-fitting and reduce computational load. We apply a two-stage procedure to make the feature selection. Stage 1 is the statistical analysis stage, and Stage 2 is the final feature subset selection stage using machine learning methods. First, we use the one-way analysis of variance (ANOVA) and then the rank-sum test to evaluate each feature. Redundant features are removed from the feature set according to the results. Second, the reduced feature set is divided into different classes. The performance is evaluated by a support vector machine (SVM) model with different kernels and parameters. The hill-climbing method is also applied to select the best feature subset.

#### 2.3.1. Statistics Analysis Stage

To determine the significance of each feature, ANOVA and the rank-sum test are used. We use a simple threshold (*p*-value of ANOVA < 0.05) to select positive features. Similarly, in the rank-sum test, there is a simple value (*p*-value = 1) to detect positive features. For each patient, the pair of *p*-values for each feature is obtained. If both *p*-values are positive for a feature, it is considered significant to a patient. After obtaining the *p*-values of 66 features for all patients, we set $\lambda_{\mathrm{feature}}$ as the number of positive pairs for each feature, and the maximum value of $\lambda_{\mathrm{feature}}$ is the number of processed patients’ PSG signals.

In this study, to select useful features, we set a threshold as half of the processed PSG signals. One feature is put into the selected subset if its $\lambda_{\mathrm{feature}}$ is more than the threshold. The selected features are then divided into different classes depending on the distribution of their $\lambda_{\mathrm{feature}}$ values. In the next stage, the hill-climbing algorithm is used to confirm the best feature subset with the classes.

#### 2.3.2. Support Vector Machine Selection Stage

To confirm the best feature subset, we used the SVM method to select the best classes with the most relevant information separating apnea from normal according to the classes obtained from the former stage. Initially, the features in the top class are fed to SVM models, and the performance is recorded. Then, the same is done to the next class. The process is repeated until all classes are done. The SVM algorithm is implemented with different kernels and parameters. In this stage, we compare the performance of different kernels. The kernels used in this paper are shown in Table 2.

To verify the performance, we use four measures, namely accuracy, sensitivity, specificity, and AUC (the area of ROC curve).

(1) $$\mathrm{sensitivity}=\frac{TP}{TP+FN}$$

(2) $$\mathrm{specificity}=\frac{TN}{TN+FP}$$

(3) $$\mathrm{accuracy}=\frac{TP+TN}{P+N}$$

where *P* is condition positive, *N* is condition negative, *TP* is true positive, *TN* is true negative, *FP* is false positive, and *FN* is false negative. Ideally, if the sensitivity value is 1 and the specificity value is 0, AUC has the largest value (AUC = 1).

### 2.4. Multi-Errors-Reduction Classification System

To improve the performance of apnea classification, we propose an MER classification. A classifier has its error, and the errors of different classifiers are in different fields. MER classification can combine the results of classifiers and minimise an error of a classifier using other classifiers, and provide labels more accurately. The MER classification consists of five phases. First, the selected features are fed to the MER classification system. Second, some classifiers with good performance are considered as basic learners in level-0. Third, we implement the classifier combination method as a potential solution to improve the classification performance. The basic assumption of classifier combination is that the misclassified instances of individual classifiers do not overlap, and different individual classifiers can provide different perspectives of classification. Classifier combinations may use complementary information to improve performance. Some basic classifier combination schemes include maximum voting, average voting, and weighted average voting. In this paper, we use the stacking ensemble method to conduct the classifier combination. In the last phase, a meta-learner is used to make the final prediction.

**Figure 3:**
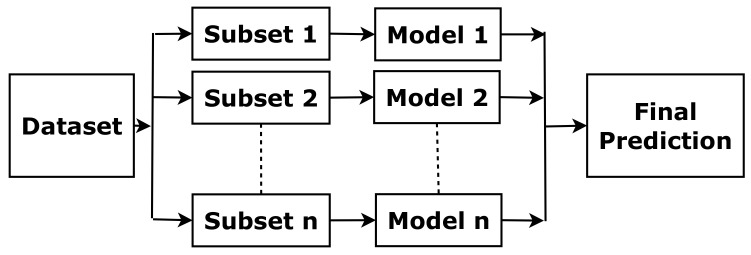
The concept of boosting.

The above method borrows a boosting concept, which is based on the idea that a combination of simple classifiers can have better performance than any of the simple classifiers alone [15]. With the same training data, a simple classifier (basic learner) is able to produce labels with a probability of error. Then, the final learner is able to minimise small error probability arbitrarily and predict labels more accurately by combining the basic learners (as illustrated in Figure 3).

In this paper, some boosting methods are used, including Gradient Boosting, CatBoost from Yandex, Light Gradient Boosting (Light GBM), and XGBoost [16]. Classic boosting methods minimise the errors by updating the weights of a training set, but Gradient Boosting uses the mistake-residual error directly. CatBoost is a kind of gradient boosting based on decision trees. “Cat” comes from “Categories”, and CatBoost can handle categorical features in a large dataset quickly. Light GBM is also an algorithm based on a decision tree, and it fits data to split the tree. It can reduce the loss and improve accuracy. The progress is made based on the leaf-wise method when growing on the same leaf. XGBoost is a decision-tree-based algorithm. It uses tree-pruning, parallel processing, handling missing values and regularization to avoid overfitting and optimise the classic boosting algorithm for enhanced performance.

**Figure 4:**
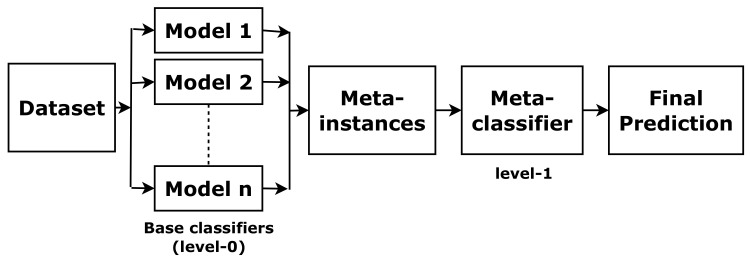
The concept of stacking.

Stacking is one of the most widely used ensemble approaches [17]. It combines predictions of base classifiers (level-0) for a meta-level (level-1) classifier. The meta-level classifier corrects the decisions of the base classifiers and predicts the final labels, as shown in Figure 4. To train the meta-level classifier, the k-fold cross-validation is used [18]. To form a meta-instance, the decisions from base classifiers are combined with the gold standard in each training fold. Then, a meta-level classifier is trained based on the meta-instances. When a new instance is classified, the outputs of the base classifiers are calculated first. Then these outputs are fed to the meta-classifier for the final results.

**Figure 5:**
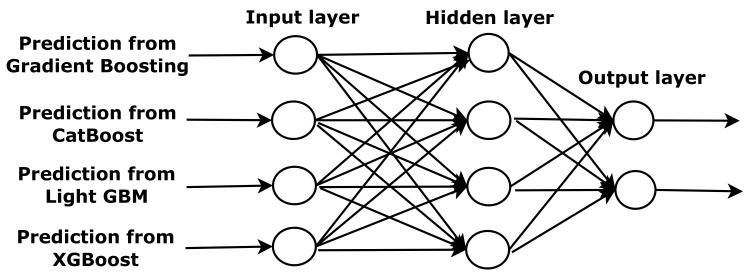
The structure of an artificial neural network.

The meta-classifier (level-1) is an ANN, extensively used for binary classification in sleep apnea studies. In Figure 5, the ANN schematic representation is shown. Each input vector is put into the input layer, and it is distributed to a neuron in the first hidden layer. Each neuron has its weight vector associated with the connections to the input vector. The neuron sums the inputs, which is processed by a non-linear activation function. The output vector of the hidden layer is multiplied by other weight vectors. The final prediction is obtained from the final layer. The number of nodes will affect the performance of the ANN. Too few hidden nodes may not fit for complex tasks. However, if the network has too many hidden nodes, the noise in the training data causes the overfitting problem [19,20].

The meta-classifier (level-1) is an ANN, which is extensively used for binary classification in sleep apnea studies. In Figure 5, the ANN schematic representation is shown. Each input vector is added to the input layer, and distributed to a neuron in the first hidden layer. Each neuron has its weight vector associated with the connections to the input vector. The neuron sums the inputs, which is processed by a non-linear activation function. The output vector of the hidden layer is multiplied by other weight vectors. The final prediction is obtained from the final layer. The number of nodes will affect the performance of the ANN. Too few hidden nodes may not fit for complex tasks. However, if the network has too many hidden nodes, the noise in the training data make causes the overfitting problem [19,20].

## 3. Results and Discussion

The experiment is divided into two parts. First, the segmentation is conducted based on event duration as it is reported by the doctor directly. It is shown in Figure 2. In this experiment part, we extract 66 features based on event duration, use the two-stage feature selection to confirm a feature subset, and then use a classifier to complete sleep apean detection. Second, the segmentation is conducted based on a common time window. In this experiment part, we extract 66 features based on a time window, use the two-stage feature selection, and then use the MER system to improve performance. Furthermore, the dataset is divided into the training set and the testing set. All feature selections and training processes were used in the training set, and the testing set was only used to evaluate performance.

### 3.1. Experiment with Event Duration

#### 3.1.1. Feature Selection

Sixty-six features were obtained by the extraction methods shown in Table 1. In this study, 1574 patients’ PSG signals were used to provide features. For each feature, there were 1574 pairs of *p*-values. $\lambda_{\mathrm{feature}}$ is the number of positive pairs for a feature. The values of $\lambda_{\mathrm{feature}}$ are shown in Table 1. For example, it can be seen that $\lambda_{\mathrm{feature}}$ of Feature 12 is 1565, which means that 1565 pairs of Feature 12 are positive. To select useful features, we set the threshold to 787 (which is half of the number of processed PSG signals). A feature is considered significant if its $\lambda_{\mathrm{feature}}$ is larger than the threshold. Finally, nineteen features were considered as significant features, which are marked with an asterisk in Table 1.

To determine the final feature subset using machine learning methods, we put the selected features into four classes {Classes A, B, C, and D} by comparing the distribution of 19 $\lambda_{\mathrm{feature}}$ values. The Classes are shown in Table 3. They were employed in the hill-climbing method in Stage 2. Kernels affect the performance of the SVM method. In this paper, we evaluate the linear (*R*), polynomial (*R* and *d*), and RBF (*R* and σ) kernels. The data were preprocessed by the under-sampled balance method, and outliers are removed. In Stage 2 of the feature selection phase, 66 features were added to SVM models with different kernels and parameters. The performance with 66 features is considered the standard. After the hill-climbing algorithm, performance was recorded and shown in Table 4. From this table, we can compare the sensitivity, specificity, and accuracy to confirm the best feature subset. The performance is bold if accuracy is more than 70%.

From Table 4, under all features, the SVM model with RBF, σ = 25, *R* = 0.2 had a sensitivity of 92.26% and a specificity 63.75% is considered as the standard. From the stability perspective, we can see that Class ABC and Class ABCD are better than Class A, Class AB, and all features. Class ABCD was more stable than Class ABC, but Class ABC had the best accuracy (79.06%). Thus, AUC was used to evaluate Class ABC and Class ABCD. A comparison of AUC under different feature subsets is shown in Table 5. The AUC of Class ABCD was better than the AUC of Class ABC. It can be found that Class ABCD had better effectiveness and robustness. Thus, Class ABCD is considered as the feature subset. Features 6, 7, 8, 10, 11, and 12 were extracted from the SaO${}_{2}$ signals, Features 23 and 26 were extracted from the abdominal signals, and Features 35, 43, 44, 45, 46, 47, 48, 49, 50, 51, and 65 were extracted from the ECG signals.

#### 3.1.2. Classification

Classification methods include the SVM algorithm, the decision tree algorithm, the k-nearest neighbour algorithm, the random forest algorithm, the extra trees algorithm, the linear discriminant analysis algorithm, and the logistic regression algorithm. The results of each classifier are shown in Table 6 and the 19 selected features are the inputs. The performance of each classifier was evaluated by sensitivity, specificity, accuracy, and AUC. The SVM method gave the highest performance (accuracy = 81.68%, sensitivity = 97.05%, specificity = 66.54%, and AUC = 81.79%).

The sensitivity of different classification methods was around 85.00%, as shown in Table 6, which means these methods are able to classify apnea events. However, the specificity was just about 68.00%, which means that normal events are not so detected by the classification methods. To improve specificity, we checked the performance of each testing set and compared the difference between the sets with good and bad results. For example, it can be found that the No. 1537 patient had 98.18% accuracy, 100% specificity, and 96.42% sensitivity, while the No. 1490 patient only had 80.52% accuracy, 61.45% specificity, and 100% sensitivity. The duration of all normal events of the No. 1537 patient was more than 60 s, and the normal events were classified. On the other hand, the normal events of the No. 1490 patient that had the 30 s duration were not labelled as normal episodes. It is found that classifiers show poor performance when they predict normal events whose duration is less than 60 s.

### 3.2. Experiment with a Time-Window Algorithm

#### 3.2.1. Feature Selection

Based on the result that normal events with a duration of less than 60 s are not able to be classified, the time-window method was used. The length of the time window was 60 s. As mentioned in the feature extraction methods, 66 features were extracted from the ECG, SaO${}_{2}$, airflow, thoracic, and abdominal signals in Table 7. Before the feature selection stage, the balanced and cleaned data were used. The statistical analysis results of $\lambda_{\mathrm{feature}}$ are shown in Table 7. Features 1–12 are from the SaO${}_{2}$ signals, features 13–22 are extracted from the airflow recordings, features 23–28 are extracted from the abdominal recordings, features 29–34 are extracted from the thoracic signals and features 35–66 are extracted from the ECG signals.

The hill-climbing algorithm was used to identify the most discriminative features. Initially, the single-best feature was picked according to the largest $\lambda_{\mathrm{feature}}$ value. In Table 7, the single-best feature was found to be feature 8 comp ($\lambda_{\mathrm{feature}}$ = 1325) from the SaO${}_{2}$ signal. It was added to the SVM method, and the performance was obtained. The next best feature was then added to the SVM method, and the performance obtained. This process was repeated until all features were added, and the best feature subset was determined by comparing all the obtained performances. Considering 66 features as inputs, the classification performance is presented in Table 8. The sensitivity was found to be 92.22%, the specificity was 81.03%, the accuracy was 88.76%, and the AUC was 86.61%. These results suggest that the performance with 66 features can be considered as the gold standard. The feature subset with similar or better performance than the gold standard was considered as the final feature subset since it can hold good performance and reduce training time. Based on the results of the hill-climbing algorithm, the best feature subset contains 48 features marked with an asterisk in Table 7, which achieves a maximum accuracy of 88.80%, with a good sensitivity of 91.95%, a specificity of 81.82%, and an AUC of 86.89%. These features are extracted from the ECG, SaO${}_{2}$, airflow, thoracic, and abdominal signals. Overall, the classifier discriminates OSA well when it is trained with the 48 features.

#### 3.2.2. Multi-Error-Reduction Classification

**Figure 6:**
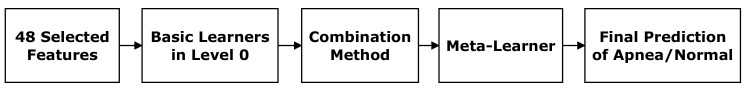
Basic components of the multi-error-reduction classification system.

To improve the performance of apnea classification, we propose an MER classification, as shown in Figure 6, which consists of five phases. First, the selected features are fed to the MER classification system. Second, some classifiers with good performance are used as basic learners in level-0. The third phase is the classifier combination method, which potentially improves the classification performance. We use the stacking ensemble method to do the classifier combination. Finally, a meta-learner is used to provide final predictions.

It is one of the crucial tasks to determine the basic learners in the MER classification system. Classification methods are used to provide results, including the SVM algorithm, Gradient Boosting, CatBoost, Light GBM, and XGBoost. The results of each classifier are shown in Table 9 with 48 selected features. From Table 9, it can be seen that the boosting methods have better performance than other classifiers. The best performance (accuracy = 90.71%) is obtained from CatBoost, with a specificity of 89.00% and a sensitivity of 91.94%. The four boosting methods are considered as the basic learners in level-0. After applying the stacking ensemble method, an ANN is used as the meta-learner. After the training, the ANN has one input layer, one hidden layer with four nodes, and one output layer. This ANN is used to provide the final prediction.

In the 60 s time-window experiment, the results of the MER classification system (accuracy = 94.66%, sensitivity = 96.37%, specificity = 90.83%, and AUC = 93.60%) have higher performance than the results from the four basic learners. Compared with the SVM model shown in Table 6, the MER classification system improves the accuracy from about 89% to 94.66%, and there is an increase in the specificity from 80% to 90.83%. The MER classification system is also used in the event duration experiment. The results of the MER system show an accuracy of 85.14%, a sensitivity of 94.96%, a specificity of 75.45%, and an AUC of 85.21%. Table 6 shows the results of other classifiers, and the MER classification system outperforms these classifiers.

We also compare our results with the results in other papers. Study [10] shows a 92.73% accuracy. In Study [9], accuracy, sensitivity, specificity are 88.13%, 84.26%, and 92.27%, respectively. In Study [2], the ROC curve analysis shows AUC, sensitivity, and specificity of 93.70%, 85.65%, and 85.92%, respectively. Our results are greater than their results, and it means the MER classification system uses multi-domain features from multi-bio signals and an ensemble system to achieve better performance. It is the potential to be used in actual doctor diagnoses and helps doctors reduce workload.

### 3.3. Discussion

In the experiment with the event duration, we found that Class ABCD is the feature subset with high discrimination, which includes 19 features. The most widely used index to diagnose OSA is that the oximetry value is less than a certain threshold or the cumulative time spent is below a threshold. In oximetry values, sudden downturns and recoveries affect the frequency band. Feature 12 is the mean of PSD within the 0.016–0.05 Hz frequency range (mean_PSD${}_{0.016/0.05}$). Feature 6 is the mean of the window (mean), Feature 7 is the number of zero-cross in the window (NumZC), and Feature 8 is the complexity (comp). Features 10 and 11 are the time spending above and below the 98% maximum (Bel98 and Abo98). Feature 12 reflects the change of the frequency band, and Features 6, 7, 8, 10, and 11 reflect the change of sudden downturns and recoveries in the time domain. In the abdominal signals, Feature 23 is the summation of the absolute window (sum_abs), and Feature 26 is the mean in the 80–100 Hz frequency range (mean_PSD${}_{80/100}$). These features are related to the active abdominal muscles during sleep apnea events. Patients with OSA show beat-to-beat variation at lower heart rates relative to healthy subjects during apnea events. The lower heart rate and the multi-domain changes lead to the selected ECG features. Feature 35 is the number of pairs of adjacent RR intervals where the later RR interval is more than 50 ms than the previous one (NN50_RR). Features 43 and 44 are spectral spread (SS) and spectral decrease (SD), respectively. Features 45–51 are Shannon’s entropy (entropy_D1 to entropy_D7) using seven detail coefficient levels. Feature 65 is the maximum of the diagonal matrix of kPCA (max_dia_kPCA).

The time-window segmentation method is used because there is no duration parameter in the actual data. The 48 features are related to the bio-physiological criteria. In the experiment with the event duration, only 19 features from the ECG, SaO${}_{2}$, and abdominal signals are put into the feature subset, and classifiers can offer good performance. However, in the experiment with the time-window method, more features were added to the feature subset, and the airflow and thoracic signals also provided some selected features. Classifiers have to fit the larger feature subset and achieve similar performance in this experiment because there is no detection of the duration of each event.

The performance of boosting methods is better than other classic machine learning methods. The reason is that the classic machine learning methods usually use the training data once, but the boosting methods repeatedly use the training data with different weights to obtain some basic classifiers. In each iteration of the boosting algorithm, the weight of each instance of the training data is estimated by the accuracy of the previous classifiers. Thus, this algorithm is able to focus on instances that are incorrectly detected. In this way, the boosting methods are able to process complex multi-domain features from different bio-signals. Figure 6 demonstrates the basic components of the MER classification system. The basic assumption of classifier combination is that the misclassified instances of individual classifiers do not overlap, and different individual classifiers can provide different perspectives for classification. The classifier combination can use complementary information to improve the performance.

## 4. Conclusions

The aim of this study is to construct an apnea classification system to detect apnea events. To achieve this aim, we design the apnea classification system, which consists of three parts: the multi-domain feature extractions, the hybrid feature selection, and the MER classification system. Multi-bio signals from PSG recordings are used to generate features, and the PSG signals are collected from the SHHS database. We adopt 1574 patients’ PSG recordings from the database. The feature extraction algorithms include time-domain, frequency-domain, and non-linear analysis.

In the experiment with the event duration, we obtain 19 selected features from different domains. These features are extracted from the ECG, SaO${}_{2}$, and abdominal signals. They reflect the change of bio-physiological signals of OSA. With the 19 features, an SVM model is used as the classifier and provides good performance (accuracy = 81.68%, sensitivity = 97.05%, specificity = 66.54%, and AUC = 81.79%). We find that classifiers do not predict the normal events of shorter than 60 s perfectly to give good specificity results. Thus, we conduct another experiment with the time-window method.

In the experiment with a time-window segmentation, 66 features are extracted from the ECG, SaO${}_{2}$, airflow, thoracic, and abdominal signals. The length of the time window is set at 60 s. After the feature selection stage, 48 features are selected from the five kinds of bio-signals. The SVM model shows good performance (accuracy = 88.80%, sensitivity = 91.95%, specificity = 81.82%, and AUC = 86.89%) with 48 features. In the MER classification system, four basic classifiers are used. They are Gradient Boosting, CatBoost, Light GBM, and XGBoost. The meta-learner is realised as an ANN. The combination method is the stacking method. The system offers a higher performance (accuracy = 94.66%, sensitivity = 96.37%, specificity = 90.83%, and AUC = 93.60%).

## Figures and Tables

**Table 1:** Feature selection results in the experiment with event duration using statistical analysis (Features 1–12 from the SaO${}_{2}$ signals, features 13–22 from the airflow signals, features 23–28 from the abdominal signals, features 29–34 from the thoracic signals, and features 35–66 from the ECG signals; four dashed lines divide the table into five parts according to the kind of signals); * denotes selected best feature subset.

Feature	λ ${}_{\mathrm{feature}}$	Feature	λ ${}_{\mathrm{feature}}$
1. med	581	34. sum_PSD${}_{80/100}$	341
2. MM2	748	35 * . NN50_RR	1432
3. kur	651	36. SDSD_RR	351
4. var	582	37. tSD_RR	563
5. min	661	38. std_RR	330
6 *. mean	1414	39. var	678
7 *. NumZC	997	40. kur	671
8 *. comp	1215	41. mean_RR	637
9. SD${}_{1}$	774	42. CV_EDR	168
10 *. Bel98	1509	43 *. SS	1477
11 *. Abo98	831	44 *. SD	1178
12 *. mean_PSD${}_{0.016/0.05}$	1565	45 *. entropy_D1	1497
13. mean	136	46 *. entropy_D2	1505
14. med	149	47 *. entropy_D3	1522
15. std	167	48 *. entropy_D4	1529
16. mean_PSD${}_{0/0.1}$	427	49 *. entropy_D5	1527
17. mean_PSD${}_{0.4/0.5}$	417	50 *. entropy_D6	1529
18. mean_D1	12	51 *. entropy_D7	1497
19. mean_D2	17	52. var_D1	606
20. mean_D3	18	53. var_D2	645
21. mean_A3	131	54. var_D3	668
22. comp	635	55. var_D4	700
23 *. sum_abs	1475	56. var_D5	660
24. std_abs	474	57. var_D6	626
25. mean	590	58. var_D7	610
26 *. mean_PSD${}_{80/100}$	947	59. WSD_RR	350
27. mean_D2	31	60. max_PSD${}_{0.03/0.5}$	352
28. mean_D1	26	61. mean_PSD${}_{10/20}$	711
29. sum	23	62. mean_PSD${}_{80/100}$	586
30. med	446	63. SCrC_3_RR	392
31. std	428	64. SCrC_4_RR	336
32. mean	16	65 *. max_dia_kPCA	1526
33. var	363	66. RP_2_PC	558

**Table 2:** List of kernel functions used in SVM.

Kernel Parameters	Mathematical Formula
Linear	K(x${}_{i}$,x${}_{j}$) = x${}_{i}$,x${}_{j}$
Polynomial	K(x${}_{i}$,x${}_{j}$) = (x${}_{i}$,x${}_{j}$+1)${}^{d}$ *d* is the degree of polynomial
Radial Basis Function (RBF)	K(x${}_{i}$,x${}_{j}$) = $e^{-{∥x_{i}-x_{j}∥}^{2}/2\sigma^{2}}$

**Table 3:** Feature classes via the distribution of λ${}_{\mathrm{feature}}$ in the experiment with event duration.

Feature No.	Class A 1526–1574	Class B 1491–1525	Class C 1301–1490	Class D 787–1300
SaO${}_{2}$	12	10	6	7 8 11
Airflow				
Abdominal			23	26
Thoracic				
ECG	48 49 50 65	45 46 47 51	35 43	44

**Table 4:** Sensitivity (%), specificity (%), and accuracy (%) based on the hill-climbing method using SVM models with different kernel functions and parameters in the experiment with event duration.

Kernels	*R*	Class A			Class AB			Class ABC			Class ABCD			All Features		
		Sen	Spe	Acc	Sen	Spe	Acc	Sen	Spe	Acc	Sen	Spe	Acc	Sen	Spe	Acc
RBF σ = 1	0.2	99.31	40.84	70.07	98.94	50.94	74.94	98.85	54.06	76.46	98.15	56.91	77.53	93.99	7.25	50.62
	1	99.12	43.10	71.11	98.90	51.87	75.39	98.68	54.92	76.80	98.24	57.88	78.06	93.70	11.66	52.68
	10	98.82	47.96	73.39	98.72	54.12	76.42	98.49	56.21	77.35	97.61	59.98	78.80	93.14	14.15	53.64
RBF σ = 5	0.2	99.06	44.87	71.96	99.09	46.42	72.75	99.15	45.85	72.50	97.58	59.99	78.78	91.76	62.07	76.92
	1	99.12	45.08	72.10	99.01	46.28	72.65	99.34	46.54	72.94	97.21	60.36	78.79	91.39	63.25	77.32
	10	99.27	40.08	69.68	99.08	48.99	74.04	98.63	54.41	76.52	97.82	58.69	78.26	89.41	65.43	77.42
RBF σ = 25	0.2	99.96	19.78	59.87	99.84	31.44	65.64	99.80	33.55	66.67	99.63	43.77	71.70	92.26	63.75	78.00
	1	99.96	24.15	62.06	99.75	35.53	67.64	99.74	37.24	68.49	98.71	49.18	73.94	91.11	64.49	77.80
	10	99.41	39.48	69.45	99.26	42.35	70.81	99.34	42.87	71.10	96.19	60.95	78.57	91.49	64.44	77.96
Poly *d* = 2	0.2	98.68	45.45	72.07	98.55	52.64	75.60	98.62	54.84	76.73	97.58	59.63	78.60	14.01	91.35	52.68
	1	98.79	45.43	72.11	98.47	52.70	75.58	98.48	55.95	77.21	97.75	59.07	78.41	11.99	91.43	51.71
	10	98.09	49.56	73.83	94.10	56.01	75.06	98.13	57.59	77.86	47.75	64.68	56.21	1.41	98.98	50.20
Poly *d* = 3	0.2	99.70	33.62	66.66	3.73	96.74	50.24	0	99.42	49.71	0	99.48	49.74	0	1	50.00
	1	95.91	38.44	67.18	1.02	99.65	50.34	0	99.42	49.71	0	99.84	49.97	0	1	50.00
	10	99.35	40.68	70.01	21.01	89.92	55.47	0	99.99	49.99	0	1	50.00	0	1	50.00
Poly *d* = 4	0.2	0	1	50.00	3.67	95.92	49.80	0	99.26	49.63	0	99.98	49.99	0	1	50.00
	1	0	99.99	49.99	0	99.92	49.96	0	99.97	49.98	0	99.95	49.98	94.24	7.21	50.72
	10	0	1	50.00	1	00.73	50.36	0	99.93	49.96	0	99.94	49.97	0	1	50.00
Linear	0.2	99.92	20.30	60.11	99.67	35.00	67.34	99.46	43.94	71.70	96.01	61.00	78.50	90.41	64.69	77.55
	1	99.91	20.31	60.11	99.65	35.05	67.35	99.01	46.86	72.94	95.80	61.00	78.40	90.62	64.67	77.65
	10	99.92	20.29	60.10	99.76	34.75	67.26	97.60	60.52	79.06	96.30	60.96	78.63	33.31	82.37	57.84

**Table 5:** AUC (%) obtained from SVM models with different kernels and parameters using Class ABC and ABCD in the experiment with event duration.

Kernels	*R*	Class ABC	Class ABCD
RBF σ=1	0.2	76.45	77.53
	1	76.80	78.06
	10	77.35	78.79
RBF σ=5	0.2	72.50	78.78
	1	72.94	78.78
	10	76.52	78.25
RBF σ=25	0.2	66.67	71.70
	1	68.49	73.94
	10	71.10	78.57
Poly *d* = 2	0.2	76.73	78.60
	1	77.21	78.41
	10	77.86	56.21
Poly *d* = 3	0.2	49.71	49.74
	1	49.71	49.92
	10	49.99	50.00
Poly *d* = 4	0.2	49.63	49.99
	1	49.98	49.97
	10	49.96	49.97
Linear	0.2	71.70	78.50
	1	72.93	78.40
	10	79.06	78.63

**Table 6:** Performance of each classifier with the selected features in the experiment with event duration.

Classifiers	Acc (%)	Sen (%)	Spe (%)	AUC (%)
SVM	81.68	97.05	66.54	81.79
Random Forest	81.60	85.27	77.98	81.62
Decision Tree	76.78	75.41	78.13	76.77
Extra Trees	81.35	85.25	77.50	81.38
K-Neighbors	78.28	84.03	72.61	78.32
Logistic Regression	81.28	96.18	66.60	81.39
Linear Discriminant	73.80	88.69	59.13	73.91

**Table 7:** Feature selection results in the experiment with time-window using statistical analysis (features 1–12 from the SaO${}_{2}$ signals, features 13–22 from the airflow signals, features 23–28 from the abdominal signals, features 29–34 from the thoracic signals, and features 35–66 from the ECG signals; four dashed lines divide the table into five parts according to the kind of signals); * denotes selected best feature subset.

Feature	λ ${}_{\mathrm{feature}}$	Feature	λ ${}_{\mathrm{feature}}$
1 *. med	542	34 *. sum_PSD${}_{80/100}$	603
2 *. MM2	1036	35 *. NN50_RR	497
3 *. kur	508	36 *. SDSD_RR	520
4 *. var	1032	37 *. tSD_RR	553
5 *. min	1079	38 *. std_RR	551
6 *. mean	1079	39 *. var	564
7 *. NumZC	572	40 *. kur	596
8 *. comp	1325	41 *. mean_RR	583
9 *. SD${}_{1}$	837	42 *. CV_EDR	534
10 *. Bel98	480	43 *. SS	570
11 *. Abo98	693	44 *. SD	561
12 *. mean_PSD${}_{0.016/0.05}$	662	45 *. entropy_D1	554
13. mean	54	46 *. entropy_D2	580
14. med	253	47 *. entropy_D3	600
15 *. std	422	48 *. entropy_D4	588
16 *. mean_PSD${}_{0/0.1}$	427	49 *. entropy_D5	535
17. mean_PSD${}_{0.4/0.5}$	54	50 *. entropy_D6	587
18 *. mean_D1	378	51. entropy_D7	138
19. mean_D2	151	52 *. var_D1	334
20 *. mean_D3	345	53. var_D2	329
21. mean_A3	324	54 *. var_D3	410
22 *. comp	394	55. var_D4	265
23. sum_abs	332	56. var_D5	293
24. std_abs	198	57. var_D6	311
25. mean	213	58 *. var_D7	333
26. mean_PSD${}_{80/100}$	331	59. WSD_RR	119
27. mean_D2	282	60 *. max_PSD${}_{0.03/0.5}$	399
28 *. mean_D1	516	61 *. mean_PSD${}_{10/20}$	470
29 *. sum	516	62 *. mean_PSD${}_{80/100}$	615
30 *. med	602	63. SCrC_3_RR	78
31 *. std	618	64. SCrC_4_RR	70
32 *. mean	671	65 *. max_dia_kPCA	578
33 *. var	574	66 *. RP_2_PC	615

**Table 8:** Two best performances in hill-climbing iterations with 48 and 66 features.

Features	Acc (%)	Sen (%)	Spe (%)	AUC (%)
48 kinds of features	88.80	91.95	81.82	86.89
A total 66 features	88.76	92.22	81.03	86.61

**Table 9:** Performance of each classifier with the 48 selected features in the 60 s experiment.

Classifiers	Acc (%)	Sen (%)	Spe (%)	AUC (%)
SVM	88.80	91.95	81.82	86.89
Gradient Boosting	90.60	93.23	86.94	90.08
CatBoost	90.71	91.94	89.00	90.47
Light GBM	90.34	91.88	88.20	90.04
XGBoost	90.55	91.73	88.90	90.32

## Data Availability

The data presented in this study are openly available at https://sleepdata.org/datasets/shhs.

## Abbreviations

The following abbreviations are used in this manuscript:

OSA	Obstructive Sleep Apnea
MER	Multi-Error-Reduction
ANOVA	Analysis of Variance
SVM	Support Vector Machine
ANN	Artificial Neural Network
SHHS	Sleep Heart Health Study
AHI	Apnea–Hypopnea Index
PSG	Polysomnography
ECG	Electrocardiogram
EEG	Electroencephalogram
EOG	Electrooculogram
EMG	Electromyogram
AF	Airflow
AB	Abdominal
TH	Thoracic
SaO${}_{2}$	Oxygen Saturation
HRV	Heart Rate Variability
EDR	ECG-Derived Respiration
PSD	Power Spectral Density
WSD	Wavelet Spectral Density
PCA	Principal Component Analysis
AUC	Area Under The ROC Curve
RBF	Radial Basis Function kernel
kNN	k-Nearest Neighbour Algorithm
Light GBM	Light Gradient Boosting
Sen	Sensitivity
Spe	specificity
*FN*	False Negative
*TN*	True Negative
*N*	Condition Negative
*FP*	False Positive
*TP*	True Positive
*P*	Condition Positive

## References

[1] Punjabi N.M. (2008). The epidemiology of adult obstructive sleep apnea. Proc. Am. Thorac. Soc..

[2] Rolon R.E., Larrateguy L.D., Di Persia L.E., Spies R.D., Rufiner H.L. (2017). Discriminative methods based on sparse representations of pulse oximetry signals for sleep apnea–hypopnea detection. Biomed. Signal Process. Control..

[3] Huang W., Guo B., Shen Y., Tang X. (2017). A novel method to precisely detect apnea and hypopnea events by airflow and oximetry signals. Comput. Biol. Med..

[4] Vaquerizo-Villar F., Álvarez D., Kheirandish-Gozal L., Gutiérrez-Tobal G.C., Barroso-García V., Crespo A., del Campo F., Gozal D., Hornero R. (2018). Utility of bispectrum in the screening of pediatric sleep apnea-hypopnea syndrome using oximetry recordings. Comput. Methods Programs Biomed..

[5] Li K., Pan W., Li Y., Jiang Q., Liu G. (2018). A method to detect sleep apnea based on deep neural network and hidden markov model using single-lead ECG signal. Neurocomputing.

[6] Mostafa S.S., Baptista D., Ravelo-García A.G., Juliá-Serdá G., Morgado-Dias F. (2020). Greedy based convolutional neural network optimization for detecting apnea. Comput. Methods Programs Biomed..

[7] ElMoaqet H., Kim J., Tilbury D., Ramachandran S.K., Ryalat M., Chu C.H. (2020). Gaussian mixture models for detecting sleep apnea events using single oronasal airflow record. Appl. Sci..

[8] Li X., Ling S.H., Su S. (2020). A hybrid feature selection and extraction methods for sleep apnea detection using bio-signals. Sensors.

[9] Bahrami M., Forouzanfar M. (2022). Sleep apnea detection from single-lead ECG: A comprehensive analysis of machine learning and deep learning algorithms. IEEE Trans. Instrum. Meas..

[10] Wang Y., Xiao Z., Fang S., Li W., Wang J., Zhao X. (2022). BI-Directional long short-term memory for automatic detection of sleep apnea events based on single channel EEG signal. Comput. Biol. Med..

[11] Morillo D.S., Rojas J.L., Crespo L.F., León A., Gross N. (2009). Poincaré analysis of an overnight arterial oxygen saturation signal applied to the diagnosis of sleep apnea hypopnea syndrome. Physiol. Meas..

[12] Otero A., Félix P., Barro S., Zamarrón C. (2012). A structural knowledge-based proposal for the identification and characterization of apnoea episodes. Appl. Soft Comput..

[13] Gutiérrez-Tobal G.C., Alonso-Álvarez M.L., Álvarez D., del Campo F., Terán-Santos J., Hornero R. (2015). Diagnosis of pediatric obstructive sleep apnea: Preliminary findings using automatic analysis of airflow and oximetry recordings obtained at patients’ home. Biomed. Signal Process. Control.

[14] De Chazal P., Heneghan C., Sheridan E., Reilly R., Nolan P., O’Malley M. (2003). Automated processing of the single-lead electrocardiogram for the detection of obstructive sleep apnoea. IEEE Trans. Biomed. Eng..

[15] Ferreira A.J., Figueiredo M.A. (2012). Boosting algorithms: A review of methods, theory, and applications. Ensemble Machine Learning.

[16] Chen T., Guestrin C. Xgboost: A scalable tree boosting system. Proceedings of the 22nd ACM SIGKDD International Conference on Knowledge Discovery and Data Mining.

[17] Wolpert D.H. (1992). Stacked generalization. Neural Netw..

[18] Bagui S.C. (2005). Combining Pattern Classifiers: Methods and Algorithms.

[19] Basheer I.A., Hajmeer M. (2000). Artificial neural networks: Fundamentals, computing, design, and application. J. Microbiol. Methods.

[20] Haykin S. (1994). Neural Networks: A Comprehensive Foundation.

